# Glomerular filtration barrier dysfunction in a self-limiting, RNA virus-induced glomerulopathy resembles findings in idiopathic nephrotic syndromes

**DOI:** 10.1038/s41598-020-76050-0

**Published:** 2020-11-05

**Authors:** Christian Nusshag, Alisa Stütz, Stefan Hägele, Claudius Speer, Florian Kälble, Christoph Eckert, Thorsten Brenner, Markus A. Weigand, Christian Morath, Jochen Reiser, Martin Zeier, Ellen Krautkrämer

**Affiliations:** 1 Department of Nephrology, Heidelberg University Hospital, Im Neuenheimer Feld 162, 69120 Heidelberg, Germany; 2 Department of Pathology, Heidelberg University Hospital, Heidelberg, Germany; 3 Department of Anesthesiology and Intensive Care Medicine, University Hospital Essen, Essen, Germany; 4 Department of Anesthesiology, Heidelberg University Hospital, Heidelberg, Germany; 5 Department of Internal Medicine, Rush University Medical Center, Chicago, IL USA

**Keywords:** Podocytes, Glomerular diseases, Viral infection

## Abstract

Podocyte injury has recently been described as unifying feature in idiopathic nephrotic syndromes (INS). Puumala hantavirus (PUUV) infection represents a unique RNA virus-induced renal disease with significant proteinuria. The underlying pathomechanism is unclear. We hypothesized that PUUV infection results in podocyte injury, similar to findings in INS. We therefore analyzed standard markers of glomerular proteinuria (e.g. immunoglobulin G [IgG]), urinary nephrin excretion (podocyte injury) and serum levels of the soluble urokinase plasminogen activator receptor (suPAR), a proposed pathomechanically involved molecule in INS, in PUUV-infected patients. Hantavirus patients showed significantly increased urinary nephrin, IgG and serum suPAR concentrations compared to healthy controls. Nephrin and IgG levels were significantly higher in patients with severe proteinuria than with mild proteinuria, and nephrin correlated strongly with biomarkers of glomerular proteinuria over time. Congruently, electron microcopy analyses showed a focal podocyte foot process effacement. suPAR correlated significantly with urinary nephrin, IgG and albumin levels, suggesting suPAR as a pathophysiological mediator in podocyte dysfunction. In contrast to INS, proteinuria recovered autonomously in hantavirus patients. This study reveals podocyte injury as main cause of proteinuria in hantavirus patients. A better understanding of the regenerative nature of hantavirus-induced glomerulopathy may generate new therapeutic approaches for INS.

## Introduction

The clinical significance of RNA virus-induced kidney diseases is highlighted by the alarming incidence of acute kidney injury (AKI) and proteinuria in patients with coronavirus disease 2019 (COVID-19)^1,2^. Tubular and especially glomerular cells such as podocytes have recently been identified as target cells of severe acute respiratory syndrome-coronavirus 2 (SARS-CoV-2)^3,4^. Of note, up to 65% of these patients show proteinuria on admission and both AKI and proteinuria are associated with poor clinical outcomes^1,5^. Puumala hantavirus (PUUV) is another RNA virus with a similar renal cell tropism^6,7^. In contrast to the life-threatening hantavirus-induced cardiopulmonary syndrome in North America, the European PUUV strain solely result in a mild form of hemorrhagic fever with renal syndrome (HFRS)^6^. The latter is characterized by rather unspecific, flue-like symptoms such as fever, limb pain, headache, diarrhea, nausea and abdominal discomfort, and about 73% of patients need inpatient treatment^8,9^. The characteristic AKI with thrombocytopenia and proteinuria occurs in 83% of cases, of which 3–5% require renal replacement therapy^8^. Pulmonary and hemorrhagic complications are rather minor and as a unique phenomenon, kidney function and proteinuria usually recover autonomously in all patients^8–10^. The typical renal histopathology in light microscopy is characterized by an acute tubular-interstitial nephritis with no or only minor glomerular changes described^11,12^. However, a transient but significant proteinuria is observed, reaching the nephrotic range in up to one third of patients^13^. Though, markers of glomerular proteinuria are detected, the exact pathogenesis and origin remains elusive^13,14^. Some authors even discuss tubular proteinuria or endothelial dysfunction as potential sources^15^. In summary, it is still unclear whether and to what extent proteinuria can be explained by a compromised podocyte and glomerular filtration barrier (GFB) integrity.

Furthermore, there are idiopathic nephrotic syndromes (INS) in which focal segmental glomerulosclerosis (FSGS) and minimal change disease (MCD) are the key findings in histological analyses^16,17^. Recently, podocyte injury and subsequent foot process effacement has been suggested as unifying pathomechanisms in INS, with the extent of podocyte injury/loss alone determining whether typical FSGS lesions occur^17^. However, the exact pathomechanisms have yet to be defined. Both immune-based drivers and circulating factors are discussed as pathogenic trigger factors^16,17^. In this context, the soluble urokinase plasminogen activator receptor (suPAR) is a proposed circulating risk factor in various human diseases associated with (nephrotic) proteinuria such as FSGS, diabetic and cardiovascular disorders, and viral infections including human immunodeficiency virus (HIV), SARS-CoV-2 and PUUV^18–25^. Published, evidence supports that suPAR is involved in a signaling pathway leading to podocyte injury, foot process effacement and proteinuria^18,26,27^. Interestingly, the same histopathological features are described in case studies of RNA virus-induced diseases such as HIV-associated nephropathy (HIVAN), COVID-19 and HFRS^4,23,28,29^.

In the present study we hypothesized that PUUV infection leads to a specific podocyte injury and subsequent proteinuria, which may resemble findings seen in INS^17,30^ and eventually other RNA virus-induced glomerular diseases. We therefore analyzed urinary nephrin excretion, as a marker for podocyte injury in relation to markers of glomerular (e.g. immunoglobulin G [IgG]) and tubular (α1-microglobulin [α1-MG]) proteinuria along with plasma levels of suPAR. Light and electron microscopic studies were performed to assess changes of GFB morphology.

## Results

### Patient characteristics

The patient characteristics are summarized in Table 1. All patients suffered from AKI and typical flu-like symptoms at the time of admission (Table S2). One patient required renal replacement therapy due to volume overload and uremia. The median days post onset of first symptoms (dpo) to hospital admission was 6.5 days. In contrast to patients with moderate creatinine-normalized proteinuria (protein-to-creatinine ratio, PCR) at the time of admission, patients with severe PCR were hospitalized earlier in terms of dpo, were longer hospitalized and showed significantly higher maximum SCr levels, maximum leukocyturia and lower minimum platelets counts. SCr on admission, baseline kidney function, laboratory inflammation parameters, hematocrit, weight gain from baseline body weight and minimum/maximum levels of urea, serum albumin, hemoglobin and extent of hematuria were comparable between both groups.

**Table 1:** Characteristics of 26 patients with acute hantavirus infection.

	All patients (n = 26) Median (Range)	Moderate PCR (≤ 2485 mg/gCr, n = 13) Median (Range)	Severe PCR (> 2485 mg/gCr, n = 13) Median (Range)	p-value
Age [years]	36.5 (18–62)	40 (18–61)	33 (23–62)	0.572
Sex [male/female]	19/7	9/4	10/3	0.658
Dpo [days]	6 (3–14)	7 (6–14)	6 (3–8)	**0.006**
Los [days]	5 (3–15)	4 (3–8)	6 (4–15)	**0.006**
Body weight gain [kg]	4.2 (0.1–12.8)	4.2 (0.10–12.8)	3.1 (0.9–10.1)	0.878
**Laboratory parameters**				
SCr baseline	0.88 (0.65–1.83)	0.88 (0.68–1.05)	0.87 (0.65–1.83)	0.626
SCr on admission [mg/dL]	4.52 (1.38–10.40)	4.57 (1.93–7.84)	4.47 (1.38–10.40)	0.626
SCr max [mg/dL]	5.1 (1.93–18.02)	4.71 (1.93–8.46)	5.71 (2.57–18.02)	**0.029**
Urea max [mg/dL]	88.5 (38.0–221.0)	82.0 (38.0–221.0)	123.0 (59.0–210.0)	0.293
Serum albumin min [g/L]	35.4 (22.7–42.4)	36.3 (30.2–43.4)	34.8 (22.7–37.7)	0.209
CRP max [mg/L]	59.5 (4.2–151.3)	60.2 (4.2–139.8)	59.7 (17.2–151.3)	0.939
Hemoglobin min [g/dL]	12.3 (10.1–14.5)	12.2 (10.6–14.5)	12.7 (10.1–13.7)	0.472
Hematocrit min [L/L]	0.35 (0.30–0.44)	0.35 (0.31–0.44)	0.35 (0.30–0.40)	0.736
Platelets min [G/L]	126.5 (21.0–291.0)	138.0 (38.0–291.0)	96.0 (21.0–154.0)	**0.021**
Leukocytes max [G/L]	10.1 (5.0–14.5)	10.0 (5.4–11.8)	10.4 (5.0–14.5)	0.209
Hematuria max [cells/µL]	20.0 (3.0–1013.0)	19.0 (3.0–252.0)	21.0 (6.0–1013.0)	0.572
Leukocyturia max [cells/µL]	37.5 (5.0–265.0)	22.0 (5.0–130.0)	48.0 (10.0–256.0)	**0.045**
ACR max [mg/gCr]	1,592.9 (13.8–29,759.2)	425.2 (13.8–1,413.0)	4,177.0 (1,772.7–29,759.2)	** < 0.0001**
PCR max [mg/gCr]	2,930.6 (42.2–50,219.7)	642.9 (42.2–2,873.6)	8,394.0 (2,987.6–50,219.7)	** < 0.0001**

ACR = albumin-to-creatinine ratio, CRP = C-reactive protein, dpo = days post onset of first symptoms, gCr = gram creatinine, Los = length of hospital stay, max = maximum, min = minimum, PCR = protein-to-creatinine ratio, SCr = serum creatinine.

Bold values are statistically significant for p < 0.05.

### Light and electron microscopy in acute hantavirus infection

**Figure 1:**
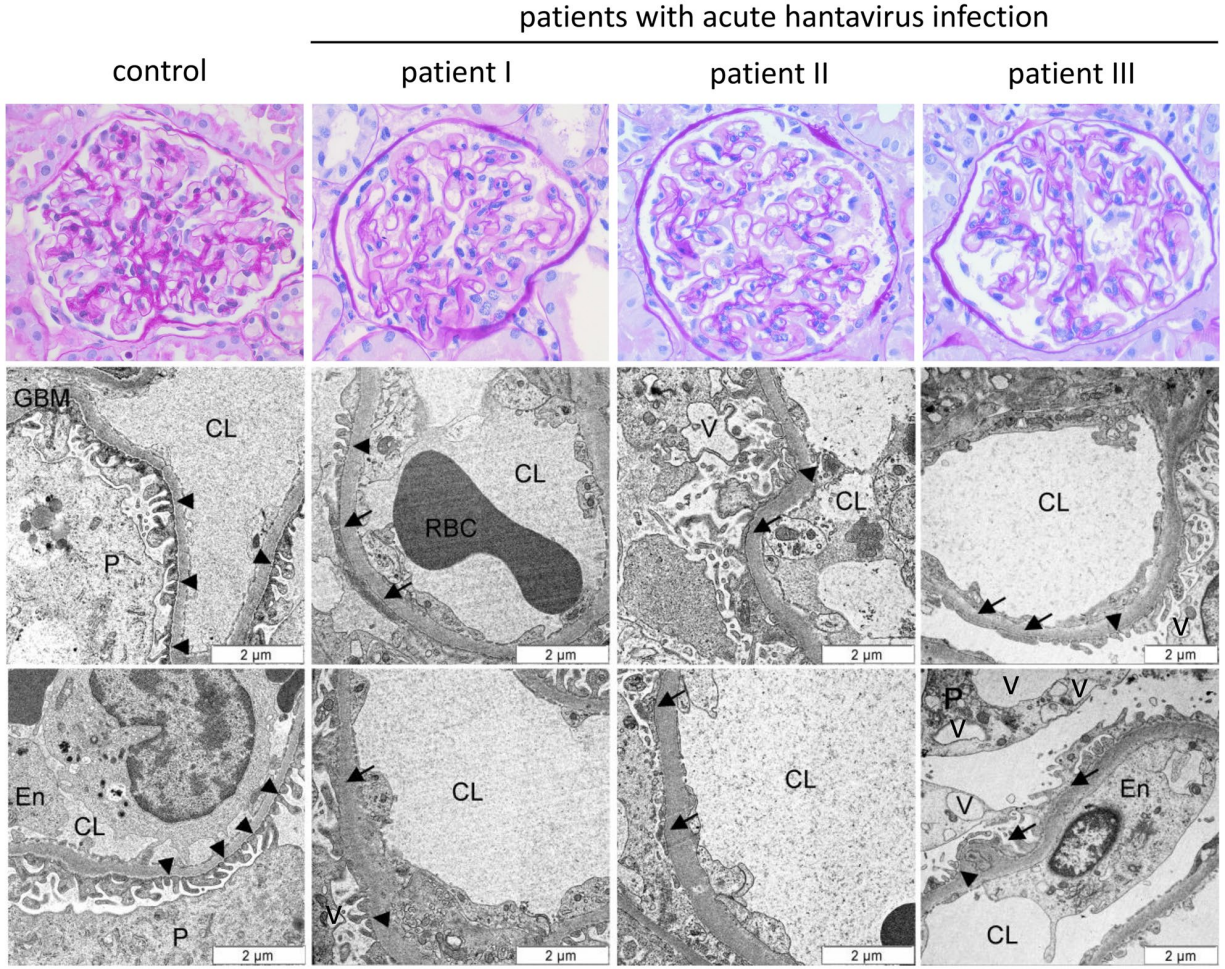
Light and electron microscopy of the glomerular filtration barrier in three patients with acute hantavirus infection and one healthy control. The glomerular filtration barrier of three patients with acute hantavirus infection (Heidelberg biopsy register) and one healthy control (living kidney donation) were analyzed by HE staining and transmission electron microscopy. Arrowheads indicate intact podocyte foot processes; arrows show effacement of podocyte foot processes. CL = capillary lumen, En = endothelium, GBM = glomerular basement membrane, P = podocyte, RBC = red blood cell, V = vacuole.

**Figure 2:**
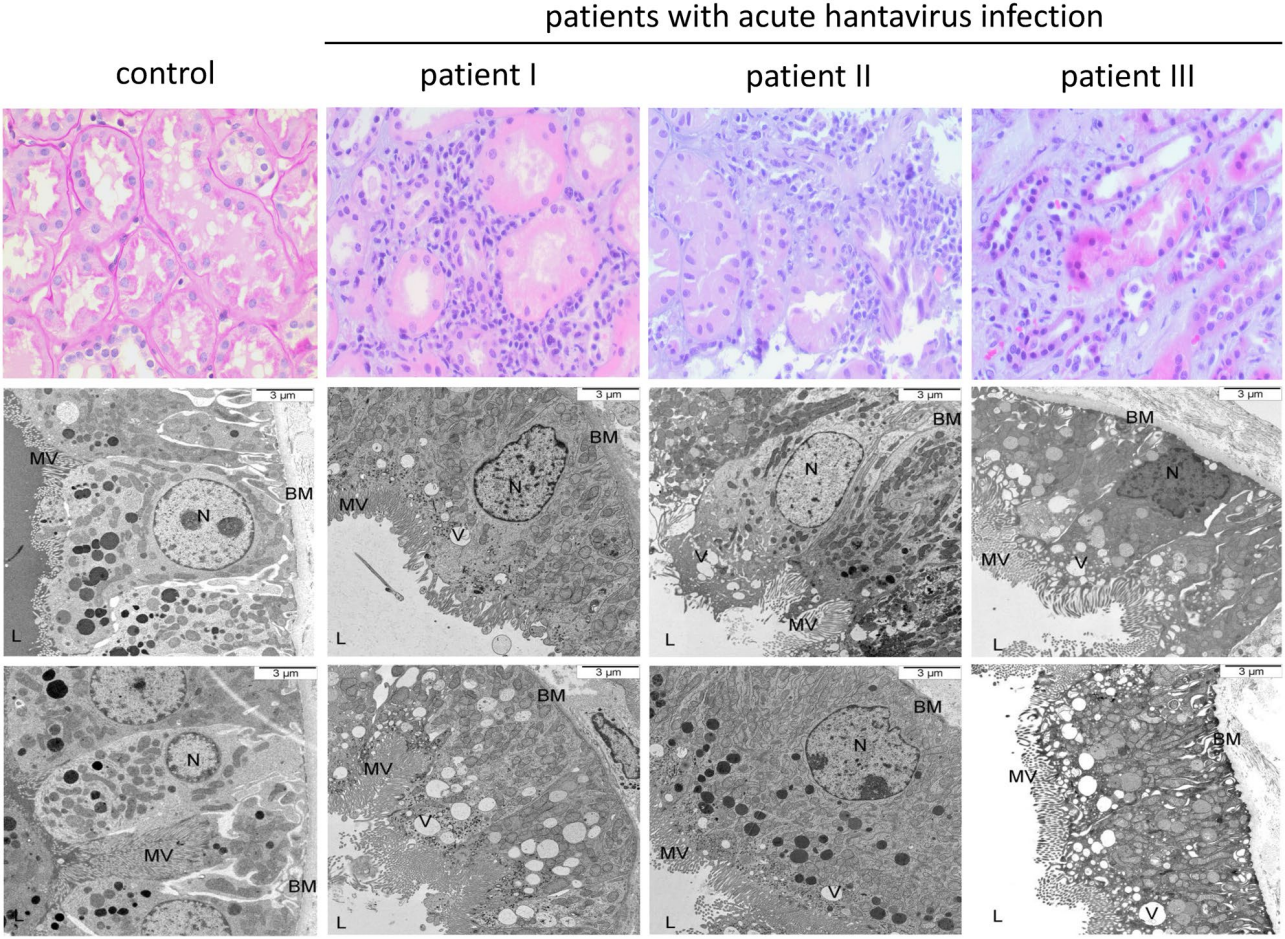
Light and electron microscopy of proximal tubular cells in three patients with acute hantavirus infection and one healthy control. Proximal tubular cells of three patients with acute hantavirus infection (Heidelberg biopsy register) and one healthy control (living kidney donation) were analyzed by HE staining and transmission electron microscopy. BM = basement membrane, L = lumen, MV = microvilli, N = nucleus, V = vacuole.

At the time of biopsy, hantavirus patients I and III had severe proteinuria of 11.2 g/d and 3570.2 mg/gCr and SCr values of 7.70 mg/dL and 4.37 mg/dL, respectively. In contrast, proteinuria in patient II had already recovered at the time of biopsy, but SCr was still elevated at 11.95 mg/dL. Light microscopy of kidney biopsy specimens from the three presented patients with acute hantavirus infection showed no glomerular pathologies such as immune cell infiltration, sclerotic lesions or mesangial hypercellularity (Fig. 1), but the characteristic picture of tubular interstitial nephritis in the renal medulla (Fig. 2). Electron microscopy of glomeruli revealed enlarged visceral podocytes, a focal foot process effacement together with a mild thickening of the glomerular basement membrane (GBM) and vacuolization of podocytes in hantavirus patients (Fig. 1, Figure S1). Immune deposits or further ultrastructural changes were not present. Mean GBM widths were 367.3 nm (± 69.6), 504.9 nm (± 74.1), 482.5 nm (± 92.6) and 685.2 nm (± 133.8) for the control and hantavirus biopsy samples I, II and III, respectively. Maximum podocyte foot process width was highest in proteinuric patients I and III with 2064.2 nm and 2301.0 nm and significantly lower in patient II and control with 1998.3 nm and 1885.5, respectively. However, due to the focal nature of the foot process effacement, mean podocyte width did not differ between patients and control (Table S3). Compared to the control, EM analysis of proximal tubular cells showed relevant subcellular lesions indicated by severe apical cytoplasmic vacuolization (Fig. 2). Interestingly, these changes were predominantly observed in the two patients with severe proteinuria at the time of biopsy (hantavirus patient I and III).

### Biomarker levels on admission

**Figure 3:**
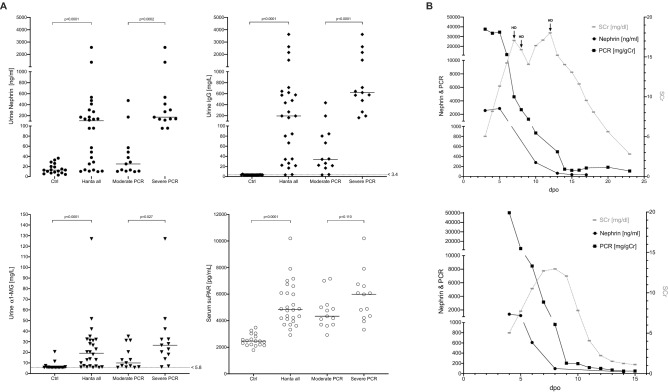
**(A)** Admission values of urinary nephrin, immunoglobulin G (IgG), α1-microglobulin (α1-MG) and serum soluble urokinase plasminogen activator receptor (suPAR) levels in relation to the extent of total proteinuria (protein-to-creatinine ratio, PCR) in patients with acute hantavirus infection (n = 26) and age and gender-matched, healthy controls (n = 18). Horizontal lines represent upper range limits of normal values when available. **(B)** Exemplary urinary nephrin and PCR courses of two hantavirus patients in relation to serum creatinine. gCr = gram creatinine, HD = hemodialysis, PCR = protein-to-creatinine ratio, dpo = days post onset of first symptoms, SCr = serum creatinine.

On admission, patients suffering from hantavirus infection showed significantly increased urinary nephrin, IgG, α1-MG and serum suPAR levels compared to healthy controls (Fig. 3A). When further dividing hantavirus patients according to the severity of PCR on admission, patients with severe PCR showed significantly higher median nephrin and IgG levels compared to patients with moderate PCR. Remarkably, an almost dichotomous distribution of urinary biomarkers of a defective GFB (nephrin and IgG) was observed when moderate and severe PCR were compared on admission, indicating substantial differences in permeability of the glomerular slit diaphragm at that specific time point. A trend towards higher suPAR levels was also seen in patients with severe proteinuria. Correlation analyses between serum suPAR levels, maximum SCr and SCr levels within the first 48 h showed no significant correlations, whereas a significant positive correlation was found for serum suPAR levels and levels of urinary nephrin, PCR, ACR and IgG (Table S4).

The normalization of nephrin and IgG levels to urinary creatinine excretion led to similar results (Figure S2). In contrast, the previous significant difference of absolute α1-MG levels between patients with moderate and severe PCR disappeared after creatinine normalization (Figure S2).

### Urine biomarker course during hantavirus infection

Though, urinary biomarker levels decreased in both groups over time, patients with severe PCR showed significantly higher levels of nephrin, IgG, ACR and PCR during the first 48 h after admission (Table 2). The greatest absolute differences were seen for urinary biomarkers that indicate a defective glomerular barrier such as nephrin, IgG, ACR. At the same time, only minor absolute differences were observed for the tubular proteinuria marker α1-MG.

**Table 2:** Urine biomarker course over 48 h after hospital admission.

Biomarker	Day	Moderate PCR (Range)	Severe PCR (Range)	p-value
Nephrin [ng/mL]	Adm (n = 26)	25.06 (9.85; 475.62)	172.70 (95.49; 2,571.03)	**0.0002**
	24 h (n = 24)	14.30 (7.49; 440.76)	109.64 (14.43; 1,162.99)	**0.007**
	48 h (n = 21)	12.59 (4.50; 119.16)	52.14 (10.73; 2,874.48)	**0.035**
IgG [mg/L]	Adm (n = 26)	34.10 (3.40; 434.00)	619.00 (162.00; 3,620.00)	** < 0.0001**
	24 h (n = 24)	13.00 (3.70; 265.00)	337.50 (5.00; 2,090.00)	**0.001**
	48 h (n = 21)	4.30 (3.70; 103.00)	80.10 (7.10; 2,760.00)	**0.001**
α1-MG [mg/L]	Adm (n = 26)	10.00 (5.80; 35.10)	26.70 (6.20; 127.00)	**0.027**
	24 h (n = 24)	9.15 (5.40; 54.80)	17.90 (8.60; 76.80)	0.094
	48 h (n = 21)	8.90 (5.40; 46.00)	18.00 (6.70; 153.00)	**0.020**
ACR [mg/gCr]	Adm (n = 26)	319.94 (12.23; 1,048.01)	4117.32 (1,772.65; 29,759.18)	** < 0.0001**
	24 h (n = 24)	100.39 (13.81; 634.56)	2113.47 (28.23; 21,126.05)	** < 0.0001**
	48 h (n = 25)	61.92 (11.55; 124.74)	335.30 (26.57; 20,803.10)	**0.001**
PCR [mg/gCr]	Adm (n = 26)	497.90 (30.59; 1,982.69)	8,392,95 (2,987.55; 50,219.72)	** < 0.0001**
	24 h (n = 24)	189.53 (51.78; 1,179.63)	4,133.69 (143.27; 33,462.55)	** < 0.0001**
	48 h (n = 25)	102.31 (40.41; 308.82)	689.94 (114.10; 34,510.80)	** < 0.0001**

Adm = day of admission, ACR = albumin-to-creatinine ratio, α1-MG = α1-microglobulin, gCr = gram creatinine, IgG = immunoglobulin G, PCR = protein-to-creatinine ratio.

Bold values are statistically significant for p < 0.05.

When analyzing urinary nephrin concentrations over time in individual patients and in relation to the dpo instead of time after admission, urinary nephrin and PCR levels decreased almost in parallel. Interestingly, the start of normalization of urinary nephrin and PCR levels preceded the first decline of SCr by 48–72 h. Furthermore, in patients with an available urine sample at the time of PCR normalization, the normalization of urinary nephrin levels tended to precede the normalization of PCR levels. Figure 3B shows two exemplary biomarker courses of patients with acute hantavirus infection. In a next step, we analyzed the course of dpo-synchronized PCR values between patients with moderate and severe PCR in the entire cohort (Table 3). Patients with severe PCR showed significantly higher PCR levels up to dpo 8 compared to patients with moderate PCR, indicating a higher renal disease severity between both groups at the same dpo. Due to the self-limiting character of the hantavirus disease and subsequent autonomous recovery of the GFB in both groups, no differences in PCR levels were seen beyond dpo 8 (Table 3). The morphology and time course of PCR slopes within both groups was comparable, but the origin of the slope started at higher PCR levels in patients with severe proteinuria, especially before dpo 8.

**Table 3:** Comparison of proteinuria in relation to the days post onset of first symptoms.

Dpo	Moderate PCR [mg/gCr]		Severe PCR [mg/gCr]		p-value
	n	Median (Range)	n	Median (Range)	
6	5	642.9 (288.7–2,873.6)	9	6,007.0 (143.3–13,534.4)	**0.042**
7	6	433.1 (95.4–1819.2)	12	3,404.0 (142.0–12,508.8)	**0.028**
8	9	196.7 (48.1–1982.7)	10	958.5 (114.1–27,041.5)	**0.028**
9	9	152.6 (28.6–1179.6)	11	373.4 (45.9–7,733.3)	0.224
10	11	140.4 (33.3–1082.0)	8	324.9 (100.7–1,209.2)	0.107
11	4	140.3 (45.3–185.5)	5	196.9 (71.8–625.2)	0.286
12	5	49.5 (28.1–161.3)	5	99.4 (47.7–147.4)	0.421
13	4	37.1 (23.2–156.6)	9	69.0 (31.0–492.6)	0.199
14	4	76.0 (30.6–188.2)	4	54.4 (41.7–146.7)	0.886

Dpo = days post onset of first symptoms, gCr = gram creatinine, PCR = protein-to-creatinine ratio.

Bold values are statistically significant for p < 0.05.

### Correlation of nephrin and laboratory parameters of hantavirus disease activity

Correlation analyses showed a strong positive correlation between urinary nephrin levels and PCR, ACR, IgG, α1-MG and C-reactive protein (CRP) levels (Table 4). The highest correlation coefficients (r) were thereby achieved between nephrin levels on admission and biomarkers of (non-selective) proteinuria. In contrast, weaker correlations where seen for SCr, especially on admission and for maximum levels. Furthermore, nephrin levels on admission showed a moderate positive association with the length of hospital stay (los) and a moderate negative association with platelet count and the time of admission in terms of dpo. Hemoglobin and leukocytes values showed no relevant correlation with urinary nephrin levels.

**Table 4:** Spearman’s correlation of urinary nephrin levels on admission with parameters of hantavirus disease activity.

Parameters	Nephrin [ng/mL] on admission		
	r	95%-CI	p-value
Dpo [days]	− 0.44	− 0.71 to − 0.05	**0.026**
Los [days]	0.53	0.17 to 0.77	**0.005**
***Urine parameters***			
**PCR [mg/gCr]**			
Adm (n = 26)	0.77	0.54 to 0.90	** < 0.0001**
24 h (n = 24)	0.79	0.56 to 0.91	** < 0.0001**
48 h (n = 25)	0.80	0.59 to 0.91	** < 0.0001**
Max (n = 26)	0.75	0.50 to 0.88	** < 0.0001**
**ACR [mg/gCr]**			
Adm (n = 26)	0.77	0.54 to 0.90	** < 0.0001**
24 h (n = 24)	0.74	0.47 to 0.88	** < 0.0001**
48 h (n = 25)	0.71	0.42 to 0.87	**0.0001**
Max (n = 26)	0.75	0.50 to 0.88	** < 0.0001**
I**gG [mg/L]**			
Adm (n = 26)	0.83	0.64 to 0.92	** < 0.0001**
24 h (n = 24)	0.77	0.53 to 0.90	** < 0.0001**
48 h (n = 21)	0.84	0.62 to 0.93	** < 0.0001**
**α1-MG [mg/L]**			
Adm (n = 26)	0.69	0.40 to 0.85	**0.0001**
24 h (n = 24)	0.60	0.25 to 0.81	**0.002**
48 h (n = 21)	0.78	0.51 to 0.91	** < 0.0001**
***Blood parameters***			
**SCr [mg/dL]**			
Adm (n = 26)	0.38	− 0.02 to 0.68	**0.055**
24 h (n = 26)	0.69	0.40 to 0.85	** < 0.0001**
48 h (n = 24)	0.76	0.50 to 0.89	** < 0.0001**
Max (n = 26)	0.70	0.42 to 0.86	** < 0.0001**
**Platelets [G/L]**			
Adm (n = 26)	− 0.67	− 0.84 to − 0.37	**0.0002**
24 h (n = 25)	− 0.67	− 0.84 to − 0.36	**0.0003**
48 h (n = 24)	− 0.64	− 0.84 to − 0.31	**0.001**
Min (n = 26)	− 0.51	− 0.75 to − 0.14	**0.008**
**Hemoglobin [g/dL]**			
Adm (n = 26)	0.23	− 0.19 to 0.58	0.259
24 h (n = 25)	0.15	− 0.28 to 0.52	0.480
48 h (n = 24)	− 0.06	− 0.46 to 0.36	0.779
Min (n = 26)	− 0.14	− 0.51 to 0.28	0.512
**Leucocytes [G/L]**			
Adm (n = 26)	− 0.01	− 0.41 to 0.39	0.959
24 h (n = 25)	0.09	− 0.33 to 0.48	0.675
48 h (n = 24)	0.17	− 0.26 to 0.54	0.428
Max (n = 26)	0.17	− 0.24 to 0.53	0.403
**CRP [mg/L]**			
Adm (n = 25)	0.43	0.03 to 0.71	**0.030**
24 h (n = 18)	0.59	0.16 to 0.84	**0.009**
48 h (n = 17)	0.63	0.19 to 0.86	**0.008**
Max (n = 26)	0.27	− 0.15 to 0.60	0.190

ACR = albumin to creatinine ratio, α1-MG = α1-microglobulin, CI = confidence interval, CRP = C-reactive protein, dpo = days post onset of first symptoms, IgG = immunoglobulin G, r = correlation coefficient, S-Alb = serum albumin, SCr = serum creatinine.

Bold values are statistically significant for p < 0.05.

## Discussion

To our knowledge, this is the first comprehensive study to investigate the role of direct podocyte damage in acute PUUV infection in vivo. Our data show a strong association between urinary nephrin levels and the extent of (non-selective) glomerular proteinuria, suggesting that hantavirus infection causes a pronounced podocyte damage and subsequent impairment of the GFB. The significant findings in electron microscopy analyses were a focal foot process effacement, podocyte vacuolization and apical tubular vacuolization (indicating massive proteinuria) which all are known as typical histopathological features in INS^16,17,31–35^. However, the pathomechanical role and underlying mechanisms of the observed podocyte vacuolization need further clarification. While differences between patients with moderate and severe proteinuria were preserved for urinary nephrin and IgG levels after normalization to urinary creatinine excretion, differences in α1-MG levels were no longer present. This further supports the idea that proteinuria in HFRS is predominantly from glomerular origin. Furthermore, when individual patients were analyzed, the normalization of urinary nephrin levels tended to precede the normalization of proteinuria. The highest correlation coefficients (r) were achieved between urinary nephrin levels on admission and PCR, ACR and IgG levels within 48 h. Both observations suggest urinary nephrin levels as an early indicator of GFB dysfunction and a direct pathophysiological connection between the current impairment of GFB integrity by hantavirus infection and the subsequent extent and clinical course of proteinuria. As a further similarity to INS^16,18^, hantavirus patients showed significantly elevated serum suPAR levels in comparison to healthy controls. suPAR levels correlated significantly with urinary nephrin, PCR, ACR and IgG levels, but not with SCr.

It was generally believed that hantaviruses predominantly infect endothelial cells, leading to capillary permeability due to a loss of cell-to-cell contacts, but without a direct cytopathic effect^6^. The deregulation of systemic angiopoietin levels^9^ and a vascular endothelial growth factor A (VEGFA)-induced downregulation of VE-cadherins accompanied by a β3 integrin-mediated dysregulation of VEGF receptor 2 (VEGF2) are suggested mechanisms^19,36–38^. Though, these findings may explain major symptoms and complications of HFRS such as capillary leakage and pulmonary edema, the exact cause of (nephrotic) proteinuria is still poorly understood. Vascular endothelial barrier dysfunction or tubular damage, as discussed by some authors^13,15^, does not adequately explain the extent of proteinuria. We have recently shown in vitro that hantaviruses additionally infect tubular epithelial cells, glomerular endothelial cells and especially podocytes, leading to disruption of cell-to-cell contacts and impaired intra-cellular integrity with rearrangements of the podocyte cytoskeleton^7,39,40^. Here, we show for the first time in vivo an interdependent relationship between ultrastructural and functional impairments of the GFB and a direct podocyte damage as indicated by increased nephrin excretion and electron microscopy. In addition, urinary nephrin, PCR, ACR and IgG levels correlated significantly with serum suPAR values. To date, one other study showed significantly elevated blood suPAR levels and their association with hantavirus disease severity but did not include nephrinuria and the extent of proteinuria in their analysis^19^. suPAR is suggested to interfere with the cross-directional signaling between the glomerular basement membrane (GBM) and podocytes, thereby affecting GFB integrity^16^. In addition, Alfano et al. recently showed that suPAR down-modulates nephrin expression in podocytes in vitro and in vivo^41^. Together, these mechanisms may disturb podocyte-GBM-interaction, where rearrangements of the podocytic cytoskeleton may result in podocyte damage, subsequent foot process effacement and GFB dysfunction with release of intercellular GFB components such as nephrin into the urine^7,16,18,39^. Further studies are required to clarify whether suPAR is a directly involved pathomechanically mediator in hantavirus-induced podocyte injury.

Nevertheless, a very unique feature in PUUV infection is that proteinuria and kidney function usually recover autonomously in all patients^8^, while therapeutic outcomes in INS are variable and autonomous recovery is rare^16,17^. Hantavirus induced proteinuria usually peaks around dpo 5 and normalizes within 1–3 weeks^13^. But, due to a varying hantavirus disease severity, the actual extent of proteinuria differs in patients^13^. The same observation applies to our cohort. Patients with severe PCR on admission still showed significantly higher PCR levels compared to patients with moderate PCR, when PCR values were matched based on the dpo instead of the time of hospital admission. SCr in turn peaks 4–5 days after the peak of proteinuria^13^. This tempts authors to claim that proteinuria predicts the severity of emerging AKI, by showing an association between glomerular proteinuria and maximum SCr levels^13^. However, it is generally accepted that SCr reflects rapid changes in renal function with a latency of at least 24–72 h (time until a new steady state is reached after a single renal insult)^42^. We therefore hypothesize that the extent of maximum renal impairment is at least in part already present at the time of maximum proteinuria but is indicated with a delay by SCr. Our results support this hypothesis by showing a stronger correlation between PCR values on admission and SCr concentrations after 24–48 h (24 h: r = 0.69, p < 0.0001; 48 h: r = 0.81, p < 0.0001) than for maximum SCr levels or SCr levels on admission (adm: r = 0.27, p = 0.1837; max: r = 0.56, p = 0.029). Other accepted markers of hantavirus disease severity such as leukocyte and platelet count, CRP and hemoglobin levels, showed weaker correlations with nephrin. This suggest GFB dysfunction once more as an independent, subcellular disease manifestation of hantavirus infection in addition to capillary leakage and AKI. Both impairment of renal function and proteinuria must probably be seen as two individual disease manifestations, the extent of each depending on the current hantavirus disease activity.

There are study limitations, which need to be addressed. First, kidney biopsy material was only available for three hantavirus patients in our database who were initially suspected to have kidney diseases other than hantavirus infection. Routine kidney biopsies could not be justified in terms of a risk–benefit analysis, due to the mostly reliable hantavirus diagnostics by serological tests and the self-limiting disease character with exclusively symptomatic therapy.

Second, the use of a creatinine-normalized description of proteinuria/albuminuria in hantavirus patients may overestimate total protein excretion relative to 24-h volume measurements because urinary creatinine excretion decreases with AKI. However, we are confident that this does not affect the results of our study, as our results were extremely consistent when absolute or creatinine-normalized proteinuria parameters were used in the analyses of glomerular proteinuria. Third, the reported minimum or maximum biomarker levels in our study may differ from the actual absolute minimum or maximum values that may have occurred prior to hospitalization.

In summary, PUUV hantavirus infection shows clinical and ultrastructural similarities to INS, but with the unique feature of an autonomous recovery. This special feature highlights the potential of further comparative studies with INS and other RNA-virus induced glomerulopathies in order to improve our understanding of regenerative mechanisms in the context of GFB dysfunctions and potential future therapeutic approaches.

## Methods

### Study design and patient population

This retrospective study was conducted at the Department of Nephrology at Heidelberg University Hospital and approved by the local Ethics Committee of the Medical Faculty of Heidelberg. All patients with acute hantavirus infection hospitalized in 2017 were included for further analyses*.* In total, 26 patients without pre-existing kidney diseases and with serologically proven, acute PUUV infection were analyzed. 18 age and gender matched volunteers served as controls (Table S1). Written informed consent was obtained from all participants. All methods or experiments were performed in accordance with relevant guidelines and regulations. The overall median creatinine-normalized proteinuria (protein-to-creatinine ratio, PCR) at the time of admission was used to categorize patients in two disease severity groups (moderate PCR vs. severe PCR): (a) moderate PCR (≤ 2485 mg/g creatinine, gCr) and (b) severe PCR (> 2485 mg/gCr). Baseline serum creatinine (SCr) was defined as the lowest value between 6 months before or after an acute hantavirus infection. Maximum PCR or SCr values were defined by the highest value measured during hospitalization or recorded in medical reports immediately prior to hospitalization.

### Data collection and laboratory methods

Patient characteristics and presented laboratory parameters such as SCr, urinary albumin-to-creatinine ratio (ACR) and PCR were obtained from medical records. Urine nephrin, IgG, α1-MG and serum suPAR levels were measured retrospectively. Nephrin and suPAR levels were quantified by enzyme-linked immunosorbent assay (ELISA) as instructed by the manufacturer (human NPHN antibody ELISA kit, Elabscience Biotech Co. Ltd, Wuhan, China; suPAR ELISA kit, R&D Systems, Minneapolis, MN, USA). Urinary IgG and α1-MG concentrations were measured in the accredited Central Laboratory of the Heidelberg University Hospital.

### Light and electron microscopy

Three biopsy samples of patients with acute hantavirus infection were analyzed. Biopsy samples (Heidelberg biopsy register) were fixed in glutaraldehyde and embedded in Epon-Araldite after post-fixation with osmium tetroxide and analyzed by transmission electron microscopy (JEM-1400, Jeol, Freising, Germany). Biopsy material from a time-zero biopsy of a living kidney allograft served as control. All analyses were performed at the Institute of Pathology (Heidelberg University Hospital, Germany). The GBM width was evaluated using 20 representative measurements per patient. To account for the focal nature of podocyte foot process effacement, foot process width was evaluated using > 100 measurements per patient or control.

### Statistical analysis

Statistical analyses were performed using SPSS Statistics 25 (IBM, Armonk, NYC, USA) and Graph Pad Prism 8 (GraphPad Software Inc., La Jolla, CA, USA). Continuous variables were presented as median (range); categorical data were presented as percentages. Mann–Whitney *U* test was used for group comparisons. Categorical variables were analyzed using Chi-square test. Correlations were assessed by using Spearman’s correlation analysis. Two-sided p values of less than 0.05 were considered statistically significant.

## Supplementary information

Supplementary Information
