# Supplementary material for: Glomerular filtration barrier dysfunction in a self-limiting, RNA virus-induced glomerulopathy resembles findings in idiopathic nephrotic syndromes
Source: Sci Rep. 2020 Nov 5;10:19117. doi: 10.1038/s41598-020-76050-0 (PMC7644703; doi:10.1038/s41598-020-76050-0)
Supplement: Supplementary file 1 — Supplementary Information [file 41598_2020_76050_MOESM1_ESM.pdf]

Supplemental material

Glomerular filtration barrier dysfunction in a self-limiting, RNA virus-induced glomerulopathy resembles findings in idiopathic nephrotic syndromes

Christian Nusshag,[1,5] Alisa Stütz,[1] Stefan Hägele,[1] Claudius Speer,[1] Florian Kälble,[1] Christoph Eckert,[2] Thorsten Brenner,[3] Markus A. Weigand,[4] Christian Morath,[1] Jochen Reiser,[5] Martin Zeier,[1] Ellen Krautkrämer,[1]

[1] Department of Nephrology, Heidelberg University Hospital, Germany
[2] Department of Pathology, Heidelberg University Hospital, Germany
[3] Department of Anesthesiology and Intensive Care Medicine, University Hospital Essen, Germany
[4] Department of Anesthesiology, Heidelberg University Hospital, Germany
[5] Department of Internal Medicine, Rush University Medical Center, Chicago, IL, USA.

Tables

Table S1: Baseline characteristics of healthy controls in comparison to hantavirus patients

	**Hantavirus patients (n=26)** Median (Range)	**Healthy controls (n=18)** Median (Range)	**p-value**
Age [years]	36.5 (18-62)	35.5 (18-63)	0.694
Sex [male/female]	19/7	13/5	0.963
Laboratory parameters			
Serum creatinine max [mg/dl]	5.1 (1.93-18.02)	0.78 (0.52-1.10)	**<0.0001**
Serum urea max [mg/dl]	88.5 (38.0-221.0)	29.0 (19.0-44.0)	**<0.0001**
Serum albumin min [g/l]	35.4 (22.7-42.4)	n.a.	
CRP max [mg/l]	59.5 (4.2-151.3)	n.a.	
Hemoglobin min [g/dl]	12.3 (10.1-14-5)	15.1 (12.4-16.80)	**<0.0001**
Hematocrit min [l/l]	0.35 (0.30-0.44)	0.44 (0.37-0.48)	**<0.0001**
Platelets min [G/l]	126.5 (21.0-291.0)	228.5 (158.0-401.0)	**<0.0001**
Leukocytes max [G/l]	10.1 (5.0-14.5)	6.41 (4.18-9.78)	**<0.0001**
ACR max [mg/gCr]	1,592.9 (13.8-29,759.2)	5.3 (2.2-13.4)	**<0.0001**
PCR max [mg/gCr]	2,930.6 (42.2-50,219.7)	43.7 (19.1-89.9)	**<0.0001**

ACR = albumin-to-creatinine ratio, CRP = C-reactive protein, gCr = gram creatinine, max = maximum, min = minimum, n.a. = not available, PCR = protein-to-creatinine ratio.

Table S2: Clinical symptoms of 26 patients with acute hantavirus infection

**Clinical symptoms**	**All patients (n=26)** Patients (%)	**Moderate PCR (n=13)** Patients (%)	**Severe PCR (n=13)** Patients (%)	p-value
Fever	23 (88.5)	11 (84.6)	12 (92.3)	0.539
Flank pain / Back pain	22 (84.6)	11 (84.6)	11 (84.6)	0.588
Abdominal pain	16 (61.5)	7 (53.8)	9 (69.2)	0.271
Headache	24 (92.3)	12 (92.3)	12 (92.3)	0.327
Nausea	21 (80.8)	10 (76.9)	11 (84.6)	0.619
Vomiting	18 (69.2)	9 (69.2)	9 (69.2)	>0.999
Diarrhea	4 (15.4)	1 (7.7)	3 (23.1)	0.277
Blurred vision	13 (50.0)	8 (61.5)	5 (38.5)	0.239

PCR = protein-to-creatinine ratio.

Table S3: Foot process width in three patients with acute hantavirus infection and one healthy control

**Statistical parameters**	**Control**	**Hantavirus patient I**	**Hantavirus patient II**	**Hantavirus patient III**
N	117	132	122	126
Mean [nm]	516.8	488.5	442.2	446.4
SD [nm]	353.3	347.6	279.1	304.8
Min [nm|	84.2	125.2	109.9	79.8
Max [nm]	1,885.5	2,064.2	1,998.3	2,301.0

Max = maximum, min = minimum, SD = standard deviation, N = number of measurements performed.

Table S4: Serum suPAR correlation analyses

Renal parameters	suPAR [pg/mL] on admission	
	r	p-value
PCR [mg/gCr]		
Adm (n=26)	0.41	**0.038**
24h (n=24)	0.42	**0.043**
48h (n=25)	0.41	**0.042**
Max (n=26)	0.44	**0.023**
ACR [mg/gCr]		
Adm (n=26)	0.50	**0.009**
24h (n=24)	0.40	0.056
48h (n=25)	0.28	0.192
Max (n=26)	0.53	**0.005**
IgG [mg/L]		
Adm (n=26)	0.51	**0.008**
24h (n=24)	0.39	0.061
48h (n=21)	0.49	**0.026**
SCr [mg/dL]		
Adm (n=26)	0.13	0.515
24h (n=24)	0.31	0.127
48h (n=21)	0.29	0.170
Max (n=26)	0.30	0.143
Nephrin [ng/mL]		
Adm (n=26)	0.45	**0.021**
24h (n=24)	0.36	0.095
48h (n=21)	0.31	0.177

ACR = albumin-to-creatinine ratio, Adm = day of admission, IgG = immunoglobulin G, PCR = protein-to-creatinine ratio, SCr = serum creatinine, suPAR = soluble urokinase plasminogen activator receptor.

Figures

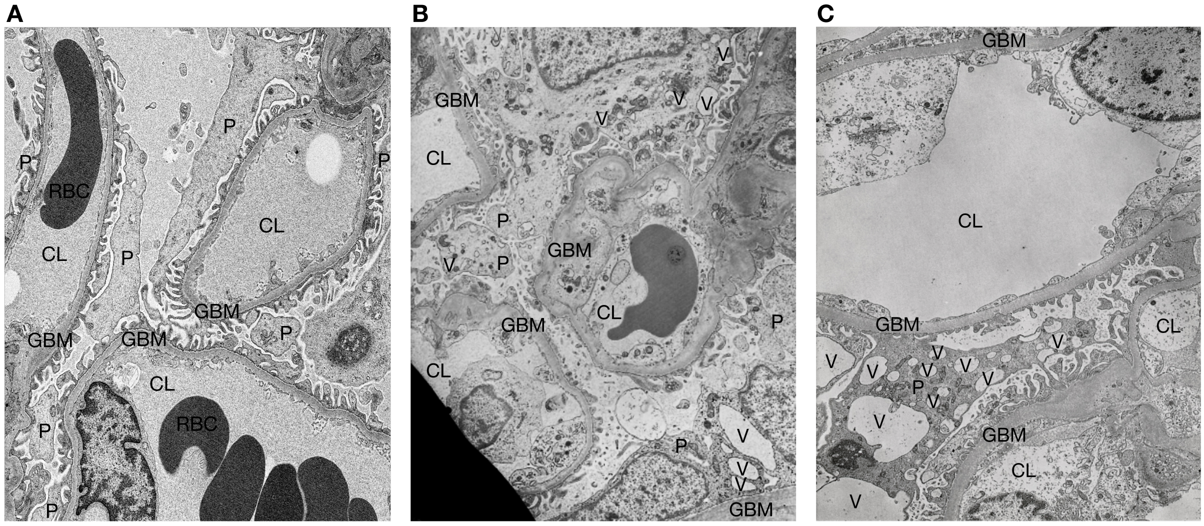

Figure S1: Exemplary electron microscopy pictures of podocyte vacuolization in patients with acute hantavirus infection. **A** healthy control (living kidney donation), **B/C** patients with acute hantavirus infection CL = capillary lumen, GBM = glomerular basement membrane, P = podocyte, V = vacuole.

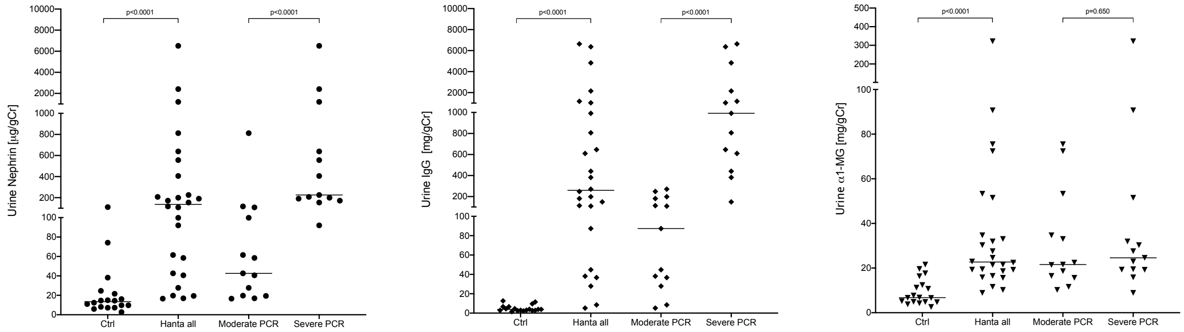

Figure S2: Admission levels of urinary nephrin, immunoglobulin G (IgG), α-1 microglobulin (α1-MG) normalized to urinary creatinine excretion and in relation to the extent of total proteinuria (protein-to-creatinine ratio, PCR) in patients with acute hantavirus infection (n=26) and age and gender-matched, healthy controls (n=18).
